# smRNAome profiling to identify conserved and novel microRNAs in *Stevia rebaudiana* Bertoni

**DOI:** 10.1186/1471-2229-12-197

**Published:** 2012-11-01

**Authors:** Vibha Mandhan, Jagdeep Kaur, Kashmir Singh

**Affiliations:** 1 Department of Biotechnology, Panjab University, Sector 14, Chandigarh, 160014, India

**Keywords:** *Stevia rebaudiana*, Next generation sequencing, Small RNAs, Conserved and novel miRNA, miRNA targets

## Abstract

**Background:**

MicroRNAs (miRNAs) constitute a family of small RNA (sRNA) population that regulates the gene expression and plays an important role in plant development, metabolism, signal transduction and stress response. Extensive studies on miRNAs have been performed in different plants such as *Arabidopsis thaliana*, *Oryza sativa* etc. and volume of the miRNA database, mirBASE, has been increasing on day to day basis. *Stevia rebaudiana* Bertoni is an important perennial herb which accumulates high concentrations of diterpene steviol glycosides which contributes to its high indexed sweetening property with no calorific value. Several studies have been carried out for understanding molecular mechanism involved in biosynthesis of these glycosides, however, information about miRNAs has been lacking in *S. rebaudiana*. Deep sequencing of small RNAs combined with transcriptomic data is a powerful tool for identifying conserved and novel miRNAs irrespective of availability of genome sequence data.

**Results:**

To identify miRNAs in *S. rebaudiana*, sRNA library was constructed and sequenced using Illumina genome analyzer II. A total of 30,472,534 reads representing 2,509,190 distinct sequences were obtained from sRNA library*.* Based on sequence similarity, we identified 100 miRNAs belonging to 34 highly conserved families. Also, we identified 12 novel miRNAs whose precursors were potentially generated from stevia EST and nucleotide sequences. All novel sequences have not been earlier described in other plant species. Putative target genes were predicted for most conserved and novel miRNAs. The predicted targets are mainly mRNA encoding enzymes regulating essential plant metabolic and signaling pathways.

**Conclusions:**

This study led to the identification of 34 highly conserved miRNA families and 12 novel potential miRNAs indicating that specific miRNAs exist in stevia species. Our results provided information on stevia miRNAs and their targets building a foundation for future studies to understand their roles in key stevia traits.

## Background

MicroRNAs (miRNAs) constitute a class of single stranded 19–24 nucleotide long non-coding RNAs that mediate endogenous gene silencing by binding to their target genes. miRNAs binds either to the open reading frame (ORF) or untranslated regions (UTRs) of target mRNAs and silencing occurs either by target cleavage or by inhibiting mRNA translation [1,2] through perfect or partial complementarity respectively [3]. miRNA feature to regulate gene expression is exemplified by their indispensable roles in critical biological and cellular events in plants including lateral root formation, leaf morphology and polarity, hormonal control, flowering time [4], cell death and apoptosis [5]. These are also involved in plant adaptation to vast array of biotic and abiotic stresses such as cold, drought, salinity, nutrient deficiency [4,6] and oxidative stress [7]. Majority of the targets of plant miRNAs were found to be transcription factors such as Squamosa-promoter Binding Protein (SBP), HD-Zip transcription factors [8] or regulatory stress response proteins like NAC domain protein and MYB proteins [7]. miRNAs along with other RNAs such as small interfering RNA (siRNA), CRISPR RNA , Piwi interacting RNA and trans-acting siRNAs (ta-siRNAs) constitute the complex smRNAome [9]. siRNAs and miRNAs are near similar in structure and function, however, several characters distinguish miRNAs from siRNAs which include the nature of precursor, conservation, target gene preference and genetic requirements for biogenesis [10].

miRNA biogenesis involves a meshwork of various enzymes. These are produced from their own transcriptional units [11] generated by stepwise processing of RNA polymerase II dependent primary miRNA transcripts (pri-miRNAs) which are subsequently capped, spliced and polyadenylated [5]. In plants, RNA binding protein DWADDLE (DDL) stabilizes pri-miRNAs for their conversion into processing centers called nuclear dicing bodies (D bodies) to stem loop precursor miRNAs (pre-miRNAs). This conversion further requires concerted assistance of Serrate (C2-H2 zinc finger protein), HYPONASTIC LEAVES1 (HYL-1, a double stranded-RNA binding protein), nuclear cap binding complex (CBC) and Dicer-like 1 (DCL-1, RNase-III class of enzymes). DCL-1 possess both Drosha and Dicer like activities instigating the initial set of both the cuts responsible for above pri- to pre-miRNA conversion and further in mature miRNA processing for releasing miRNA/miRNA* duplex in the nucleus, supposed to be predominant location of DCL-1. Hua Enhancer 1 (HEN-1), a methyl transferase, adds methyl groups to the 3^′^ ends of duplex for stabilization which is further exported in cytoplasm by HASTY (HST) proteins, plant ortholog of Exportin-5 [5]. Finally, depending on the thermodynamic stability of 5’end, miRNA strand is preferentially loaded into RNA induced silencing complex (RISC) whereas miRNA* strand is degraded. The RISC complex guides miRNA strand to its target mRNA which is then cleaved between 10^th^ and 11^th^ bases from 5^′^ end of miRNA match. The cleaved halves of target mRNA are then degraded by exoribonucleases 4 (XRN-4) and secondary siRNAs [12,13].

miRNAs were initially discovered as developmental timing regulators in *Caenorhabditis elegans*[14] and later found to be ubiquitously present in wide range of animals, plants, some viruses and in unicellular organisms such as green algae, *Chlamydomonas reinhardtii*[15]. Increasing evidences showed that miRNA repertoire of plant or animal species includes a set of conserved (ancient, abundant) and non-conserved or novel (species specific, recently evolved) miRNAs [16]. A total of 18226 entries representing hairpin precursor miRNAs, expressing 21643 mature miRNA products, across 168 species have been deposited in the publicly available online repository for miRNA sequences, miRBase Release 18 [17]. However, the biggest challenge in plants is to identify novel miRNAs and to understand their mode of action, role in various metabolic processes.

*Stevia rebaudiana* Bertoni (family Asteraceae) is one of 154 members of genus *Stevia* and one of the only two species that produce sweet stevoil glycosides [18]. *S. rebaudiana* is a perennial herb which accumulates up to 30% (w/w leaf dry weight) diterpenoid steviol glycosides (SGs) [19]. SGs are glucosylated derivatives of diterpenoid alcohol steviol and stevioside and rebaudioside A are the major SGs found in stevia. Sweetness indices of these and other related compounds ranges between 30 to 300 times higher than that of sucrose [20] and are used as non-calorific sweeteners in many countries of the world. In addition to being a sweetener, stevia has been suggested to exert beneficial effects on human health and is considered to have anti-hyperglycemic, anti-hypersensitive, anti-oxidant, anti-tumour/anti-inflammatory, immunomodulatory, antidiarrheal, antimicrobial and anti-rotavirus activities [18]. Stevioside has been reported to lower the postprandial blood glucose level of Type II diabetes patients and blood pressure in mildly hypertensive patients [21,22]. SGs biosynthesis pathway is divided into two stages; early stage wherein geranylgeranyl diphosphate (GGDP) is synthesized and late stage, which is involved in the synthesis of SGs from GGDP. SGs being important compounds of stevia, imparting medicinal properties to the plant, so the molecular studies done on this plant were mainly focussed on understanding the biosynthesis and regulation of the genes involved in SG biosynthesis. A control over the regulation of these early and late genes can help to manipulate the diterepenoid contents [23]. Further, the unraveling of sRNA guided circuitry in stevia will enhance the value of its gene and EST information and improve our ability to devise strategies to enhance certain essential features of stevia that are less amenable to functional genomics analysis leading to its enhanced nutritive value. The identification of miRNA and their targets is important not only to help us learn more about the roles of miRNAs in stevia development and physiology but also to provide a framework for further designing RNAi based experiments for regulation of gene expression in this species.

Genome of *S. rebaudiana* has not been sequenced yet and further there are only 5548 stevia expressed sequecend tags (ESTs) and 136 gene sequences available in the NCBI database. Thus, it was not possible to perform an extensive study to discover stevia miRNAs using computational analysis with this limited number of available sequences. Experimental cloning combined with computational prediction appears to be the most effective method to predict stevia miRNAs. The availability of high throughput next generation sequencing (NGS) technologies such as 454 and Illumina have further revolutionized sRNA discovery. Whereas, transcribed sequences such as ESTs has led to the identification of only conserved (abundant) miRNAs or miRNAs previously identified in other species, on the other hand, NGS provides high throughput tools to make new discoveries of additional species specific or lowly expressed miRNAs in plants irrespective of whether their genome is sequenced or not, e.g. *Arabidopsis*[16], *Oryza sativa*[24], *Populus trichocarpa*[25], *Triticum aestivum*[26], *Brachypodium distachyon*[27], *Vitis vinifera*[28], *Arachis hypogaea*[29], *Citrus trifoliate*[30] , *Carthamus tinctorius*[31] and many more. This huge list signifies that miRNAs have been extensively studied since years and the digging of smRNAome of any organism is of utmost importance to understand the sRNA mediated gene regulations and the diversity of sRNAs.

Our research aimed to examine sRNA population in leaves of *S. rebaudiana* using Illumina platform to identify conserved and novel miRNAs. The downstream analysis of sRNA data generated by genome analyzer II led to the identification of 100 conserved miRNA sequences belonging to 34 miRNA families sharing very high homology with that already known in other species. In addition, 12 putative novel miRNA families were also identified with their precursors. A scan of the *S. rebaudiana* EST and nucleotide databases with miRNA sequences revealed their putative targets. Putative targets have been predicted for 12 out of above 34 conserved miRNA families and 5 novel miRNAs using computational methods.

## Results

### Deep sequencing of sRNAs

Using 36 cycled single end sequencing by genome analyzer II [32], a total of 30,472,534 sequences were obtained from smRNAome of *S. rebaudiana.* After removing the low quality and adaptor sequences, 17,295,850 sequences were obtained. Among these sequences 15,327,722 sequences ranged from 16–28 nt in length. Further removing t/rRNAs, a total of 6,075,858 sRNA sequences were obtained with a size range of 19–24 nt (Table 1).

**Table 1:** Statistics of sequences obtained from the stevia sRNA library

**sRNA**	**Redundant reads**	**Unique reads**
Total sRNA sequenced in library	30,472,534	-
Sequences matching 3’ adaptor sequence	17,295,850	-
Sequences matching 3' adaptor sequences within size range specified: (16–28 nt)	15327722	2509190
Filter valid miRNA size range: (19-24nt)	7,020,896	629987
Filter t/rRNA	6,075858	627710
Filter sequences matching miRBase-18-mature.fa	6075552	627678

Although some sRNAs were high in abundance and present thousands of times in our dataset, majority of sRNAs were sequenced only once in our experiment e.g. 2,509,190 sequences out of 15,327,722 sequences were sequenced only once (Table 1). This suggested that (1) there is a significant variation in expression of various sRNAs in stevia; (2) sRNA survey in stevia is far from being exhausted. This ultimately directs that stevia contains a large and diverse sRNA population as was the case seen in *C. trifoliate*[30].

**Figure 1:**
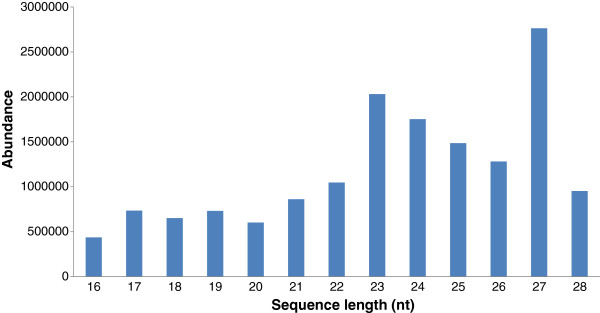
**Length distribution and abundance of sRNA sequences of *S. rebaudiana*.**

In stevia, the size of sRNAs was not evenly distributed (Figure 1). Among these sequences, the number of 23nt long sequences was unusually greater than other longer and shorter sequences and accounted for 13.25% of the total sequence number. Class of 23–25 nt has been recently reported and named as long miRNAs discovered in *Arabidopsis*[33]. This result was unlike as seen in peanut [29], cotton [34], medicago [35] and early phase maize seedlings [36] where fraction of 23 nt was very small as compared to others.

In total, 1,752,385 sequences of 24 nt length (11.43%) represented the second highest followed by 22nt sequences (6.83%) and finally 21 nt sequences (5.61%) which is the typical length of plant mature miRNAs (Figure 1). The latter trend of abundances of 24nt sequences followed by 22nt and 21nt was same as observed during seed germination in maize (36). Additionally, 27,63512 sequences with a length of 27nt were present, covering 18.02% in the stevia library. They may represent the degradation products during sRNA library construction.

### Identification of conserved stevia miRNAs

**Figure 2:**
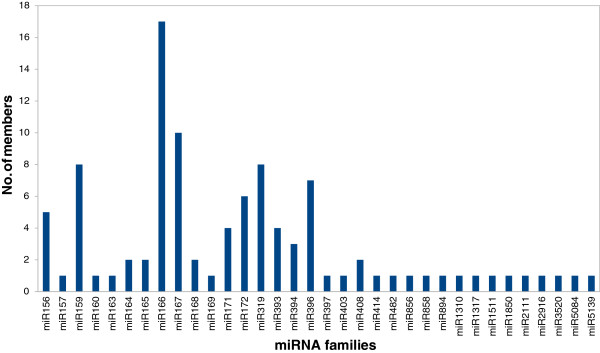
**Number of identical miRNA members in each family in *S. rebaudiana***.

The filtered sRNA dataset were compared with the currently known mature plant miRNAs in miRBASE v18 [37] using miRPROF. After multiple BLASTN searches and further sequence analysis, a total of 100 conserved miRNAs, belonging to 34 miRNA families (Table 2) were identified. Most of the miRNA families were found to be conserved in a variety of plant species e.g. using a comparative genomics based strategy homologs of miR319, miR156/157, miR169, miR165/166, miR394 and miR159 were found in 51,45,41,40,40 and 30 diverse plant species respectively [38]. Deep sequencing aids by providing an alternative way to estimate the expression profiles of miRNA genes [39]. It not only determines the abundance of various miRNA families but simultaneously distinguishes between different members of a given family. On the basis of similarity in mature miRNA sequence, these miRNA were grouped into families, with members often varying upto 1 or 2nt. Diversity of stevia miRNAs could also be found in the aspect of number of members each family contained (Figure 2).

**Table 2:** Profile and abundance of conserved miRNAs obtained from *S. rebaudiana* sRNA library

**miRNA family**	**miRNA**	**Sequence**	**Length (nt)**	miRNA* sequenced	**Reads**	**Homolog**
156/157	miR156a	UGACAGAAGAGAGUGAGCACAUC	23	N	124	bna-MIR156a
	miR156b	UGACAGAAGAGAGUGAGCAUC	21	N	124	ath-MIR156a
	miR156c	UUGACAGAAGAUAGAGAGCACAC	23	N	124	ath-MIR157c
	miR156d	UGACAGAAGAGAGUGAGCACAU	22	N	124	rco-MIR156d
	miR156e	UGACAGAAGAGAGUGAGCACUC	22	N	3	ath-MIR156e
	miR157a	UUGACAGAAGAUAGAGAGCACAC	23	N	1	ath-MIR157a
159	miR159a	UUUGGAUUGAAGGGAGCUCUAUC	23	N	11759	ath-MIR159a
	miR159b	UUUGGAUUGAAGGGAGCUCAUC	22	N	11827	ath-MIR159b
	miR159c	UUUGGAUUGAAGGGAGCUCUAAU	23	N	12411	vvi-MIR159c
	miR159d	UUUGGAUUGAAGGGAGCUCUGUC	23	N	80	sof-MIR159d
	miR159e	UUGGAUUGAAGGGAGCAUC	19	N	6	osa-MIR159e
	miR159f	UUGGAUUGAAGGGAGCUCUAAU	22	N	136	osa-MIR159f
	miR159j	UUUGGAUUGAAGGGAGCUCU	20	N	74	zma-MIR159j
	mirR159k	UUUUGGAUUGAAGGGAGAUC	20	N	74	zma-MIR159k
160	miR160a	UGCCUGGCUCCCUGUAUGCCAA	22	N	1	ath-MIR160a
163	miR163	AGAUGACUUGGAACUUCGAUC	21	N	2	ath-MIR163
164	miR164c	UGGAGAAGCAGGGUACGUGCAUC	23	N	8	osa-MIR164c
	miR164h	UGGAGAAGCAGGGCACGUAUC	21	N	2	zma-MIR164h
165	miR165a	UCGGACCAGACUUCAUCCCCCA	22	N	5	ath-MIR165a
	miR165b	UCGGACCAGGCUUCAUCCCCCUC	24	N	5	ath-MIR165b
166	miR166a	UCGGACCAGGCUUCAUUCCCCAU	22	Y	412	ath-MIR166a
	miR166b	UCGGACCAGGCUUCAUUCCAUC	23	N	398	crt-MIR166b
	miR166c	UCGGACCAGGCUUCAUUCCUCAU	23	Y	357	mtr-MIR166c
	miR166d	UCGGACCAGGCUUCAUUCCCCAC	23	Y	396	ath-MIR166d
	miR166e	UCGGACCAGGCUUCAUUCCCCA	22	Y	292	ath-MIR166e
	miR166f	UCUCGGACCAGGCUUCAUUC	20	N	379	bdi-MIR166f
	miR166g	UCGGACCAGGCUUCAUUCCUCUC	23	Y	357	osa-MIR166g
	miR166h	UCGGACCAGGCUUCAUUCCUCGA	23	N	357	osa-MIR166h
	miR166i	UCGGACCAGGCUUCAUUCCCC	21	N	291	ath-MIR166b
	miR166j	UCGGACCAGGCUUCAUAUC	19	N	291	ptc-MIR166j
	miR166k	UCGGACCAGGCUUCAUUCCU	20	N	304	sbi-MIR166k
	miR166l	UCGGACCAGGCUUCAUUCCUCAC	23	N	357	zma-MIR166l
	miR166m	UCGGACCAGGCUUCAUUCCUCC	22	N	397	zma-MIR166m
	miR166n	UCGGACCAGGCUUCAUUCCUUAU	23	N	370	ptc-MIR166n
	miR166o	UCGGACCAGGCUUCAUUCCUUCG	23	N	286	ptc-MIR166o
	miR166p	UCGGACCAGGCUCCAUUCC	19	N	1	ptc-MIR166p
	miR166q	UCGGACCAGGCUUCAUUCCUUUC	23	N	286	ptc-MIR166q
167	miR167a	UGAAGCUGCCAGCAUGAUCUGAUC	24	N	9629	ccl-MIR167a
	miR167b	UGAAGCUGCCAGCAUGAUCUAAUC	24	N	9637	bna-MIR167b
	miR167c	GAAGCUGCCAGCAUGAUCUGAUC	23	N	7862	bdi-MIR167c
	miR167d	UGAAGCUGCCAGCAUGAUCUGAU	23	N	7853	bdi-MIR167d
	miR167e	UGAAGCUGCCAGCAUGAUCUGUC	23	N	73	osa-MIR167e
	miR167f	UGAAGCUGCCAGCAUGAUCAUC	22	N	21	gma-MIR167f
	miR167g	UGAAGCUGCCAGCAUGAUCUGA	22	N	7806	gma-MIR167g
	miR167h	UGAAGCUGCCAGCAUGAUCUG	21	N	13	osa-MIR167h
	miR167i	UGAAGCUGCCAGCAUGAUCUAC	22	N	65	sbi-MIR167i
	miR167j	UGAAGCUGCCAGCAUGAUCUGC	22	N	13	osa-MIR167j
168	miR168a	UCGCUUGGUGCAGGUCGGGAAUC	23	Y	71	ath-MIR168a
	miR168b	UCGCUUGGUGCAGGUCGGGAAU	22	Y	69	ath-MIR168b
169	miR169f	UAGCCAAGGAUGACUUGCCUAUC	23	N	7	osa-MIR169f
171	miR171a	UGAUUGAGCCGUGCCAAUA	19	N	10	osa-MIR171b
	miR171b	UGAGCCGUGCCAAUAUCAUC	20	N	10	ath-MIR171b
	miR171c	UUAUAGAGCCGUGCCAAUA	19	N	10	osa-MIR171c
	miR171d	UGAUUGAGCCGUGCCAAUC	19	N	10	osa-MIR171d
172	miR172a	AGAAUCUUGAUGAUGCUGCAUUC	23	Y	20	ath-MIR172a
	miR172b	AGAAUCUUGAUGAUGCUGCAUC	22	N	20	ath-MIR172b
	miR172c	AGAAUCUUGAUGAUGCUGCAGAU	23	N	24	ath-MIR172c
	miR172d	AGAAUCUUGAUGAUGCUGCAUAU	23	N	25	osa-miR172d
	miR172e	AGAAUCUUGAUGAUGCUGCAUCA	23	N	20	tcc-MIR172e
	miR172f	AGAAUCUUGAUGAUGCUGCA	20	N	20	ath-MIR172a
319	miR319a	UUGGACUGAAGGGAGCUCCCUUC	23	N	76	ath-MIR319a
	miR319b	UUGGACUGAAGGGAGCUCCAUC	22	N	76	ath-MIR319b
	miR319c	UUGGACUGAAGGGAGCUCCCU	21	N	78	vvi-MIR319c
	miR319d	UUGGACUGAAGGGAGCAUC	19	N	23	ptc-MIR319d
	miR319e	UUGGACUGAAGGGAGCUCCC	20	N	14	ppt-MIR319e
	miR319f	UUGGACUGAAGGGAGCUCCCC	21	N	78	vvi-MIR319f
	miR319g	UUGGACUGAAGGGAGCUCCCACC	23	N	528	vvi-MIR319g
	miR319h	UGGACUGAAGGGAGCUCAUC	20	N	12	ptc-MIR319g
393	miR393a	UCCAAAGGGAUCGCAUUGAUCCC	23	N	4	ath-MIR393a
	miR393b	UCCAAAGGGAUCGCAUUGAUCCAU	24	N	4	ath-MIR393b
	miR393c	UCCAAAGGGAUCGCAUUUAUC	21	N	2	zma-MIR393a
	miR393d	UCCAAAGGGAUCGCAUUGAUC	21	N	2	ptc-MIR393d
394	miR394a	UUGGCAUUCUGUCCACCUCCAUC	23	N	1554	vvi-MIR394a
	miR394b	UUGGCAUUCUGUCCACCUCCUC	22	N	2	ath-MIR394b
	miR394c	UUUGGCAUUCUGUCCACCUCCAU	23	N	1554	vvi-MIR394c
396	miR396a	UUCCACAGCUUUCUUGAACUAUC	23	Y	23	vvi-MIR396a
	miR396b	UUCCACAGCUUUCUUGAACUUUC	23	Y	17	aqc-MIR396b
	miR396c	UUCCACAGCUUUCUUGAACUUAU	23	N	17	osa-MIR396c
	miR396d	UUCCACAGCUUUCUUGAACUUC	22	N	17	ptc-MIR396d
	miR396e	UUCCACAGCUUUCUUGAACU	20	N	29	tcc-MIR396e
	miR396f	UUCCACGGCUUUCUUGAACUGAU	23	N	32	ptc-MIR396f
	miR396g	UUCCACGGCUUUCUUGAACAUC	22	N	2	ptc-MIR396g
397	miR397b	AUUGAGUGCAGCGUUGAUGAAUC	23	N	2	bdi-MIR397b
403	miR403a	UUAGAUUCACGCACAAACUCGUC	23	N	1	ptc-MIR403a
408	miR408a	UGCACUGCCUCUUCCCUUA	19	N	1	sof-MIR408a
	miR408b	UGCACUGCCUCUUCCCUGGAUC	22	N	6	ppt-MIR408b
414	miR414	AUCAUCAUCAUCAUCAUCA	19	N	1	ath-MIR414
482	miR482a	UCUUCCCCACACCUCCCAU	19	N	1	gso-MIR482a
856	miR856*	UGAUGUUAAUUGUGGUAUC	19	Y	1	aly-MIR856
858	miR858	UUCGUUGUCUGUUCGACCUUGA	22	N	2	ath-MIR858b
894	miR894	CGUUUCACGUCAGGUUCACCA	21	N	17	ppt-MIR894
1310	miR1310	AUCGGGGGCGCAACGCCUC	19	N	2	pta-MIR1310
1317	miR1317	AAAUGAUCUUGGAGGUUAUC	20	N	3	osa-MIR1317
1511	miR1511	AACCUGGCUCUGAUACCAUC	20	N	1	gma-MIR1511
1850	miR1850	UGUUUAGUUGCCAUCAAUC	19	N	2	osa-MIR1850
2111	miR2111a	UAAUCUGCAUCCUGAGGUUUAUC	23	N	1	ath-MIR2111a
2916	miR2916	GGGGGCUCGAAGACGAUCAUC	21	N	68	peu-MIR2916
3520	miR3520	AGAGAGACUUUUGAAUUAUC	20	N	1	ahy-MIR3520
5084	miR5084	UAGAGUACUGUAGAGGAUC	19	N	1	tae-MIR5084
5139	miR5139	AACCUGGCUCUGAUACCAUC	20	N	2	rgl-MIR5139

**Figure 3:**
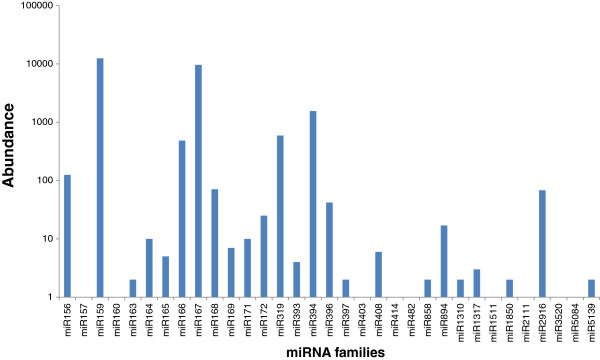
**Abundance of stevia conserved miRNA families.** Only one read was detected form miR157, 160, 403, 414, 482, 1511, 2111, 3520, 5084.

Further, miRNA families displayed significant varied abundance from each other. For e.g. majority of stevia miRNAs were sequenced less than 1000 times whereas some miRNAs were detected in low counts i.e. less than 10 times (Figure 3). Among the 34 miRNA families, miR159 proved to be largest one with highest number of sequences. In addition, miR167 and miR394 were found to have some thousands to tens of thousands of redundancies while miR319, miR166 and miR156 had more than one hundred redundancies. The remaining families were infrequently sequenced (less than 100).

A vast diversity was further seen in abundance of different members of a single family. For e.g. - as miR159a showed 11,759 sequenced clones i.e. more than 10,000 times. On the other hand, miR159e was sequenced just 6 times. As the samples were collected in the pre-flowering phase the existence of a dominant member in a miRNA family may suggest that the regulatory role of this family was performed by the dominant member in that particular developmental phase.

### Identification of novel stevia miRNAs

In this study, due to unavailability of whole genome sequence of *S. rebaudiana*, we predicted potential novel miRNAs based on bioinformatics analysis using the stevia EST and nt sequences available in NCBI database. First, we removed the sequencing tags that can be annotated as non- coding RNAs including t or rRNAs and then filtered the known miRNA sequences (Table 1) from further analysis.

A total of 6,075,552 redundant sequences survived the above filtration steps and 627,678 unique sequences were analyzed for novel miRNA prediction. A distinguishing feature of miRNAs is the ability of their pre miRNA sequences to adopt canonical stem loop hairpin structure. These unique sRNA sequences were mapped to EST and nt sequences by searching all *S. rebaudiana* sRNAs and predicting the secondary structure of sequences surrounding them (±150bp) (Additional file 1 Figure S1). We identified 12 sequences that satisfied the secondary structure and other criterions (Table 3).

**Table 3:** Novel miRNAs identified in *S. rebaudiana*

**miRNA**	**Sequence**	**Length**	**Clones**	**EST/nt accession no.**	**Strand**	**A+U content**	**Mismatch**	**miRNA start**	**miRNA end**	**EST/nt start**	**EST/nt end**	**Precursor**
Stv_1	AAACGGUGAACAAGAAGAGG	20	1	BG522782.1	Plus	55%	0	1	20	470	489	YES
Stv_2	UGUGAAGGCGGAUGACUGUAU	21	1	BG523272.1	Plus	55%	0	1	21	216	236	YES
Stv_3	UGAGAUGUUCAAUGGAAAGUAA	22	1	BG526716.1	Plus	68.2%	0	1	22	142	163	YES
Stv_4	GAGACUGAUUUCAGAACCGG	20	1	BG524295.1	Plus	50%	0	1	20	240	259	YES
Stv_5	ACGUACGAUCAUUUGGAUAAGAU	23	1	BG525786.1	Plus	65.2%	0	1	23	167	189	YES
Stv_6	UUGAACAAGUAUGGUCGUCCC	21	5	AY215182.1	Plus	52.3%	0	1	21	435	455	YES
Stv_7	UCGUGCUGUUGGGAAGUGGA	21	1	BG526771.1	Plus	42.3%	0	1	20	327	346	YES
Stv_8	CUGACGUGUCACCAUUACGGA	21	1	BG525715.1	Minus	47.6%	0	1	21	323	303	YES
Stv_9	GGUAAAGCACUGUUUCGGUGC	21	1	BG523023.1	Plus	47.6%	0	1	21	324	344	YES
Stv_10	UUGACACUUUCCCGGGACA	19	2	BG522709.1	Plus	42.1%	0	1	19	95	113	YES
Stv_11	GGCGGGCUCAAAUGACGAAUCAU	23	1	BG524443.1	Plus	47.5%	1	1	23	378	400	YES
Stv_12	CUCGCGCUUUGGUUGAAGAAC	21	1	BG526387.1	Plus	47.6%	0	1	21	266	286	YES

Corresponding hairpin loop structures are provided in supplementary information.

A sRNA is considered as a potential miRNA candidate only if it meets the following strict criteria as defined by Zhang et al. [40]; 1) the sequence could fold into an appropriate stem loop hairpin secondary structure; 2) the small RNA sits in one arm of hairpin structure; 3) no more than 6 mismatches between mature miRNA sequence and its opposite miRNA * sequence in the secondary structure; 4) no loop or break in the miRNA or miRNA * sequences; 5) predicted secondary structure has negative minimal folding free energy; 6) minimum A+U% (30-70%) as mature miRNAs contain more A+U nucleotides than G+C [41] and 7) usually begins with 5' U which is one of the characteristic feature of miRNAs.

### Target prediction of miRNAs

miRNA target prediction in plants is easier due to high and significant complementarities of miRNA- mRNA targets [42] and targets of most plant miRNAs possesses a single perfect or near perfect complementary site in coding region [43]. Assuming this to be generally the case, the *Stevia* transcript library (EST and nucleotide databases) was searched for complementarity with the sequences of identified conserved and novel miRNAs using the two target prediction web servers psRNATarget and TAPIR . Almost all the targets predicted through psRNA target server were further authenticated by TAPIR which showed the same targets. Further, TAPIR predicted additional putative targets. A deep insight into miRNA targets helps us in understanding the range of sRNA expression, regulation and their functional importance. To analyze probable sRNA targets is significant in plants because the complimentary sites of potential sRNA can exist anywhere along the target mRNA rather than at 3’UTR in case of animals [44].

We predicted target genes and putative targets were identified for 12 out of 34 conserved families (Additional file 2 Table S1) and for 5 out of 12 stevia specific or novel miRNAs (Table 4). Further, mode of action of miRNAs on targets was found to be predominantly through cleavage which is usually the case seen in plants [1,6]. Mostly multiple regulatory targets were predicted e.g. miR 414 has been predicted to have almost 70 targets in stevia EST and nucleotide transcript library. The predicted targets are mainly mRNA encoding enzymes regulating essential plant metabolic and signaling pathways which include transcription factors, elongation factors, protein kinases, heat shock proteins, F-box family proteins, catalases, peroxidases, aminopeptases, dehydrogenases, transferases, synthases, esterases, oxidoreductases etc.

**Table 4:** Putative target genes of novel miRNAs identified using psRNATarget and TAPIR programs

**Novel miRNA**	**Target Acc.**	**Tool used**	**Score**	**mfe**	**Start**	**Seed gap**	**Seed mismatch**	**Seed gu**	**Gap**	**Mismatch**	**gu**	**E**	**UPE**	**Inhibition**	**Annotation**	**e-value**
Stv_1	BG523199	psRNA/TAPIR	2.5	0.7	55	0	0	1	0	1	1	2	9.668	Cleavage	AT1G55490 [Arabidopsis thaliana]	4e-71
Stv_2	BG525025	psRNA/TAPIR	0	1	418	0	0	0	0	0	0	0	10.485	Cleavage	Phosphoglycerate kinase [*Arabidopsis thaliana*]	8e-101
Stv_7	BG526734	psRNA/TAPIR	3.5	0.78	463	0	1	0	0	1	1	3	16.578	Cleavage	Pectinacetylesterase family protein [*Arabidopsis thaliana*]	3e-108
Stv_8	BG525715	psRNA/TAPIR	2	0.89	303	0	1	0	0	0	0	1.5	16.677	Cleavage	Predicted protein [*Populus trichocarpa*]	2e-06
Stv_10	BG521995	TAPIR	3.5	0.82	594	0	1	1	0	0	1	-	---	-------	Ribulose-1,5-biphosphate carboxylase activase [*Oryza sativa*]	1e-63

**mfe**: free energy ratio; **start**: start position of the duplex on the mRNA; **seed gap**: no.of gaps in seed region; **seed mismatch**: no.of mismatches in seed region; **seed gu**: number of G–U pairs in seed region; **mismatches**: number of mismatches, **gu**: number of G–U pairs; **gap**: number of gaps introduced by bulges and loop structures; **Score**: score calculated for each miRNA–mRNA duplex taking into account all the above described parameters. **E** (Expectation): scores < 3.0 considered as a potential target of miRNA; **UPE** :Target accessibility - allowed maximum energy to unpair the target site.

## Discussion

A sudden inclination of extensive research towards investigation of plant miRNAs is due to their indispensable roles in plant development and adaption. At present more than thousand miRNA genes among diverse plant species have been identified, annotated and some of them have been well characterised [37]. But still a large number of plant species are unexplored and stevia is one of them. Till date several agrotechniques, bioproduct extraction, phytochemical, biological and toxicological studies have been carried out on *S. rebaudiana*[18]. A large flux of research activities have diverted to understand the biosynthesis and possible manipulation of diterpenoids in stevia, particularly SGs which are mainly responsible for its ecological and commercial importance. But till date no research activity is reported for exploring the miRNA repertoire in stevia. A vast survey of miRNAs in stevia will provide useful information to elucidate their physiological functions, gene regulation, biogenesis and evolutionary roles in plants.

Traditional strategies like genetic screening, microarray and bioinformatics prediction have miserably failed in species where genome sequence is not available such as stevia. The availability of high throughput NGS technologies has overshadowed this weakness by generating an accurate and comprehensive picture of smRNAome and identification of known miRNAs but also for successful discovery of novel miRNAs with high precision. It is also used as miRNA expression profiling tool to understand miRNA function in various molecular and physiological pathways. In this report, we describe the first screen for stevia miRNAs by deep sequencing with an aim of gaining insights into various roles of miRNAs. In total, 17,295,850 high quality sequences were generated from 30,472,534 raw sequences representing 57% of the total raw reads. RNA species 23, 24, 22 and 21 nt in length dominated the small RNA transcriptome with the 23 nt class being the most abundant in our library. This class of 23–25 nt has been named as long miRNAs. This finding is consistent with the working hypothesis that evolutionary change in structure of hairpin can change their affinity for DCL’s and hence the size of miRNAs. So, it is actually DCL3 which is responsible for pri-miRNA processing to generate 23 nt long miRNAs in contrast to the highly stringent DCL 1 for 20–21 nt canonical miRNAs. Further at times DCL2 can also process certain pri-miRNAs and generate 22 nt long miRNAs [33] justifying their presence.

Further a total of 30, 472, 534 raw sequences generated a profile of 34 conserved miRNA families. Our data were in good harmony with previous studies of miRNA profiling based on exhaustive sequencing of sRNA populations (e.g. In grapevine 24 million reads gave 26 known miRNA families, in peanut 6,009,541 reads gave 22 know miRNA families and in *C. trifoliate* 13, 106,573 reads gave 42 known miRNA families) [ 28, 29,30]. This probably reflects the generally accepted high level of expression reported for conserved miRNAs. Stevia miRNA families showed varied abundance from each other. Among the 34 miRNA families mir159 showed the largest number of sequenced clones which are in agreement as miR159 is also the most abundant family in *Arabidopsis*[16]. Further mir159 and mir394 with highest abundance in stevia were among the moderately expressed miRNAs in *Arachis hypogea*[29] and among the lowly expressed miRNAs in *C. trifoliate*[30]. In stevia miR156 was among the lowly expressed miRNAs but usually miR156 represents one of the highly abundant miRNA families in diverse plant species e.g. *Arachis hypogea*[29], *Brachypodium*[27] and early maize seedlings [36]. The actual reason why these miRNA families showed varied abundances in different plant species in unknown. Thus this varied abundance of miRNA families suggested that miRNA genes would be differentially transcribed at this young leaves stage.

Certain miRNA families are monocot or dicot specific which is further justified by the presence of miR403 (dicot specific miRNA) in stevia. The abundance of miR172 was 20 times low as compared to miR156 in our dataset which is consistent with the previous finding that these two miRNAs are conversely regulated [36]. Further, the largest miRNAs family size identified was miR166 that consisted of 17 members. miR156, miR159, miR167, miR319, miR396 and miR172 possessed 5, 8, 10, 8, 7 and 6 members respectively whereas other miRNA families such as miR157, miR160, miR169, miR858, miR894, miR2111 etc. had only one member detected in this library. The size of miRNAs families may be indicative of their function.

Based on the results from the deep sequencing, different family members displayed drastically different expression levels. For example, the abundance of miR159 family varied from 6 reads to 11,759 reads in the deep sequencing. This was also the case for some other miRNA families, such as miR156 (from 3 read to 124 reads) miR167 (from 13 reads to 9,637 reads) and miR394 (from 2 reads to 1,554 reads). Abundance comparisons of different members in one miRNA family may provide useful information on the role that miRNAs play in the pre-flowering stage and even importance of the dominant member at that particular stage of plant growth. On the contrary 12 novel miRNAs were predicted according the criterion mentioned before. All the 12 followed secondary structure criteria forming a stem loop structure with miRNA sequence sitting at one arm of the hairpin and negative minimal folding energy. The A+U contents of predicted miRNAs were found in the range of 42.1- 68.2%. Out of these 12 sRNAs, sequences of stv_2, 3, 6, 7 and 10 start with 5’U. Though stv_7 and stv_10 contained less than 50% A+U content but they start with U. Keeping this in view, these two sRNAs could be putative stevia miRNAs. Earlier accepted findings state that non-conserved miRNAs are usually expressed at lower levels showing a tissue or development specific pattern [30] as was seen in case of stevia where the read number of novel miRNAs was much lower than that for conserved miRNAs. Majority of them had only 1–5 sequenced clones (Table 3). This suggests miRNAs identified in stevia might represent only a meager portion of novel miRNAs due to the fact that sRNA library was constructed from young plant leaves under normal conditions.

It has been shown that plant miRNAs exhibit high degree of sequence complementarity to their targets which facilitates effective target prediction. Target genes identified seem to be associated not only with development but also with diverse physiological processes. It was found that target sites for conserved miRNAs in this plant were similar or functionally related to validated plant miRNA targets e.g. most members of the *Squamosa Promoter Binding Protein Like* (*SPL*) *transcription factor family* are targeted by miR156 in plants [8]. For instance, miR156 targets 11 of the 17 *SPL* genes in Arabidopsis. *SPLs* affect diverse developmental processes such as leaf development, shoot maturation, phase change and flowering in plants. Similarly, in stevia miR156 has been found to target *SPL*. Similarly, miR160 targets *auxin response factor 10* (*ARF*) and miR414 targets Zinc finger related protein (Additional file 2 Table S1). miR414 is predicted to regulate multiple targets including *RING/U-box domain-containing protein*, *phytochrome E*, *WD-repeat protein*, *vacuolar ATP synthase* subunit E, *SIN3 component*, *translation elongation factor*, molecular chaperone *Hsp90*, *ALF* domain class transcription factor, *programmed cell death 2 C*-terminal domain-containing protein, resistance protein, *NAC*, *DNA-damage repair* protein and several proteins involved in synthesis i.e. *60S acidic ribosomal protein* etc. Further, miR414 is predicted to have interesting stevia genes as targets like *kaurene synthase* (KS22-1), *calmodulin* and *UDP-glycosyltransferase 73E1*. *Kaurene synthase* is a vital late gene involved in steviol glycoside biosynthesis pathway. Further targets were predicted for certain more conserved miRNAs including miR166, miR167, miR319, miR 396 and miR408, miR856 and miR1310 (Additional file 2 Table S1). miRNAs regulate gene expression predominantly by cleavage (Additional file 2 Table S1) due to high complementarity of miRNA and targets.

Putative targets were also predicted for some newly identified miRNAs. Stv_1 targets one of the chaperonin_like superfamily protein whose common function is to sequester non-native proteins inside their central cavity and promote folding by using energy derived from ATP hydrolysis. On the other hand, Stv_2 targets phosphoglycerate kinase (PGK) which is a monomeric enzyme that catalyzes the transfer of the high-energy phosphate group of 1,3-bisphosphoglycerate to ADP, forming ATP and 3-phosphoglycerate. This reaction represents the first of the two substrate-level phosphorylation events in the glycolytic pathway. Stv_7 targets pectinacetylesterase family protein which is located in cell wall or membrane and exhibits carboxylesterase activity. Further, Stv_10 is predicted to target ribulose-1,5-bisphosphate carboxylase activase(P-loop NTPases) involved in diverse cellular functions as well as lupus la ribonucleoprotein or RRM (RNA recognition motif) found in proteins involved in post-transcriptional gene expression processes including mRNA and rRNA processing, RNA export, and RNA stability. Additionally, we predicted a few genes with unknown function and hypothetical genes for miRNA targeting e.g. Stv_8 (Table 4). Target of only 5 novel miRNAs were predicted speculating that rest of the miRNA may be typically present in stevia and needs further experimental work to locate their gene targets.

The above described information regarding predicted targets could be utilized efficiently to assess the regulatory roles of these novel miRNAs in stevia. Further if any of these putative novel miRNA is found to have control over the regulation of the early and late genes in steviol glycosides biosynthetic pathway as seen in case of conserved miRNA 414 can help to manipulate the diterepenoid contents.

## Conclusion

The present study provides an important glimpse of sRNA abundance and diversity in stevia. We discovered, for the first time, 34 highly conserved miRNA families and 12 novel potential miRNAs from *S. rebaudiana* using high throughput solexa sequencing of sRNAs, which indicated that species specific miRNAs exist in stevia. The isolated sRNAs may be considered as stevia putative miRNAs based on their characteristic features. These results show that regulatory miRNAs exist in nutritionally important stevia and play a role in stevia growth, development and response to stress. Further prediction of target genes adds to our understanding of mechanisms regulating their cellular function and evolution. Most importantly, this study will serve as a foundation of future research into functional roles of miRNAs and will prove to be a major breakthrough if any of these miRNA will be targeting the genes involved in steviol glycosides pathway.

## Methods

### Plant material and RNA isolation

Plants of *S. rebaudiana* were grown and maintained in Panjab University, Chandigarh, India. Young leaves were harvested by snap freezing method and stored at −80 °C till further use. Total RNA enriched with sRNA fraction was isolated using protocol developed by Ghawana et al. [45] combined with miRNAeasy spin kit (Qiagen, Germany). Briefly, 100 mg of tissue was ground with liquid nitrogen using a pestle and mortar. Powdered tissue was mixed with solution-I to make a homogeneous mixture. Solution-II was added and ground again for a while. Supernatant was extracted twice with 200μl of chloroform. To the upper aqueous phase, 1.5 volumes of 100% ethanol was added and mixed thoroughly. Then the samples were loaded onto miRNAeasy column and centrifuged. The column was washed once with wash buffer provided in the kit and on-column DNase digestion was done to remove residual DNA using the RNase-free DNase set (Qiagen, Germany). The column was washed again with wash buffer and clean RNA enriched with sRNA fragments was eluted using elution buffer.

### sRNA library preparation and sequencing

RNA samples were sent to the Microarray and Genomic Core Facility, Huntsman cancer institute, University of Utah, Salt Lake City, USA, for preparation of the sRNA library and sequencing. Briefly, sRNAs were separated on polyacrylamide gel by electrophoresis and RNA bands corresponding to the size range 20 to 30 nucleotides (nt) were purified from the gel using Illumina’s Small RNA Sample Prep Kit. The 3^′^ adapter (5^′^-UCGUAUGCCGUCUUCUGCUUGUidT-3^′^) and 5^′^ adapter (5^′^-GUUCAGAGUUCUACAGUCCGACGAUC-3^′^) were ligated to sRNAs in two steps. Adapters were designed to ligate specifically to sRNAs containing 5^′^ monophosphate and 3′ hydroxyl ends that were found in the DCL-catalyzed products [46]. Adapter ligated sRNAs were converted to cDNA and then amplified in PCR. PCR-generated DNA libraries were subjected to the 36 cycled single-end sequencing using the Genome Analyzer II (Illumina, USA).

### Bioinformatics analysis

#### Processing of raw sequences

To analyze the sRNA data, online version of the UEA sRNA toolkit [47,48] was used. Toolkit provides a package of various tools for the analysis of high-throughput sRNA data including the sequence preprocessing tool which aids in conversion of FASTQ to FASTA format, removal of adaptor sequences, extracting sequences of defined size range and their abundance calculation. The adaptor sequences were removed by finding exact matches of the 3^′^ adaptor and optional 5^′^ adaptor sequences on the raw sequences. Filter tool removed t/r-RNAs (non-coding) sequences from further analysis by comparing the t/rRNA sequences available at Rfam database, genomic tRNA database and EMBL release 95. sRNAs in the size range of 19nt to 24nt were extracted which constitutes the ideal miRNA size range.

#### Identification of conserved miRNAs

The online repository for miRNA sequences, miRBase Release 18, was used to search for known and conserved miRNAs using miRPROF (known miRNA expression profile) [47] allowing only 2 mismatches with mature miRNAs in miRBASE [17]. Parameters taken into consideration were sequences matching to viridiplantae mature miRNA v18 and further unchecked parameters including group by match signature, combine miRNA family members and combine mature and star sequences leaving all other parameters at their default settings.

#### Prediction of novel miRNAs

Stevia ESTs (5548) and nt (136) sequences were downloaded from the NCBI database. For detection of novel miRNAs of stevia, conserved miRNA sequences were removed from processing file and the rest were used to perform BLASTN searches against stevia sequences in order to obtain precursors for potential novel miRNAs. The selected EST and nt sequences which showed significant complementarity with the sRNA in the dataset were then folded into a secondary structure using RNA fold annotation tool provided in the UEA sRNA tool kit which uses Vienna RNA package to fold miRNA precursors using the minimum free energy algorithm [49] and yields a single optimal structure. The sRNA was considered to be a novel miRNA if a perfect stem loop structure was formed with sRNA sequence at one arm of the stem as well as keeping other criteria given by Meyers et al. [50] under consideration.

#### Target gene prediction

The potential targets of conserved and novel miRNAs were predicted using the psRNATarget program [51] and secondly checked with new web server called TAPIR [52], designed for the prediction of plant miRNA targets. psRNATarget program was executed with parameters at their default settings i.e. score or maximum expectation at 3, length of complementary scoring at 20, target accessibility at 25, flanking length around target 17 bp upstream and 13 bp downstream and keeping range of central mismatch leading to translational inhibition in between 9–11. Newly identified conserved and novel miRNA sequences were used as custom miRNA sequences and *S. rebaudiana* transcript library (EST and nucleotide databases) was used as custom plant database.

All predicted target genes were evaluated under the psRNA target server by scoring, and the criteria used were as follows: each G:U wobble pairing was assigned 0.5 points, each indel was assigned 2.0 points, and all other non-canonical Watson-Crick pairings were assigned 1.0 point each. The total score for an alignment was calculated based on 20 nt. When the query exceeded 20 nt length, scores for all possible consecutive 20 nt subsequences were computed, and the minimum score was considered the total score for the query-subject alignment. Because targets complementary to the miRNA 5’ end appear to be critical, mismatches other than G: U wobbles at positions 2–7 at the 5’ end were further penalized by 0.5 points in the final score [53]. Sequences were considered to be miRNA targets if the total score was less than 3.0 points.

For further validation of the above miRNA targets TAPIR was used keeping the score cutoff value (default 4) and the free energy ratio cutoff value (default 0.7) using FASTA search engine. TAPIR indicates several parameter values (names, free energy ratio, start position of the duplex on the mRNA, seed and non-seed mismatches, gaps and G–U pairs) including a full representation of the miRNA–mRNA duplex with an alignment string. Sensitivity and specificity tests for the TAPIR fast method was done with psRNA target server using various parameters. All the results showed that TAPIR with the FASTA search engine (score cutoff 4) has a higher rate of true positive, while keeping the false positives to values that are similar to those of psRNA target [54]. Once potential target mRNA sequences were obtained, BLAST was performed using these target sequence and the NCBI database as reference to predict functions of potential targets.

## Abbreviations

miRNAs: MicroRNAs; sRNA: Small RNA; ORF: Open reading frame; UTRs: Untranslated regions; SBP: Squamosa-promoter Binding Protein; ta-siRNAs: Trans-acting siRNAs; pri-miRNA: Primary miRNA; DDL: DWADDLE; D bodies: Dicing bodies; pre-miRNAs: Precursor miRNAs; HYL-1: HYPONASTIC LEAVES1; CBC: Cap binding complex; DCL-1: Dicer-like 1; HEN-1: Hua Enhancer 1; RISC: RNA induced silencing complex; XRN-4: Exoribonucleases 4; SGs: Steviol glycosides; GGDP: Geranylgeranyl diphosphate; ESTs: Expressed sequecend tags; NGS: Next generation sequencing; Nt: Nucleotide; miRPROF: Known miRNA expression profile.

## Authors’ contributions

VM carried out actual laboratory work, bioinformatics analyses of sequences and drafted the manuscript. JK has helped in experimental design, bioinformatics analysis and manuscript preparation. KS conceived, designed the experiment, associated with results interpretation, analysis and coordinated the study. All the authors have read and approved the manuscript.

## Supplementary Material

Additional file 1 **Figure S1.** Mapping of stevia novel miRNAs onto their corresponding EST/gene sequences available in database. Hairpin loop was formed and miRNA was present on one arm of loop (highlighted in red color). Sequence of miRNAs and its corresponding EST/ gene accession number is also mentioned. Click here for file

Additional file 2 **Table S1.** Target genes of conserved miRNAs identified using psRNATarget and TAPIR programs. Click here for file
