# Supplementary material for: smRNAome profiling to identify conserved and novel microRNAs in Stevia rebaudiana Bertoni
Source: BMC Plant Biol. 2012 Nov 1;12:197. doi: 10.1186/1471-2229-12-197 (PMC3502355; doi:10.1186/1471-2229-12-197)
Supplement: Additional file 1 — Figure S1. Mapping of stevia novel miRNAs onto their corresponding EST/gene sequences available in database. Hairpin loop was formed and miRNA was present on one arm of loop (highlighted in red color). Sequence of miRNAs and its corresponding EST/ gene accession number is also mentioned. [file 1471-2229-12-197-S1.doc]

**Supplementary Figure 1**: Mapping of stevia novel miRNAs onto their corresponding EST/gene sequences available in database. Hairpin loop was formed and miRNA was present on one arm of loop (highlighted in red color). Sequence of miRNAs and its corresponding EST/ gene accession number is also mentioned.

Stv_1 AAACGGUGAACAAGAAGAGG

(BG522782.1)

Stv_2 UGUGAAGGCGGAUGACUGUAU

(BG523272.1)

Stv_3 UGAGAUGUUCAAUGGAAAGUAA

(BG526716.1)

Stv_4 GAGACUGAUUUCAGAACCGG

(BG524295.1)

Stv_5ACGUACGAUCAUUUGGAUAAGAU

(BG525786.1)

Stv_6 UUGAACAAGUAUGGUCGUCCC

(AY215182.1)

Stv_7 UCGUGCUGUUGGGAAGUGGA

(BG526771.1)

Stv_8 CUGACGUGUCACCAUUACGGA

(BG525715.1)

Stv_9 GGUAAAGCACUGUUUCGGUGC

(BG523023.1)

Stv_10 UUGACACUUUCCCGGGACA

(BG522709.1)

Stv_11 GGCGGGCUCAAAUGACGAAUCAU

(BG524443.1)

Stv_12 CUCGCGCUUUGGUUGAAGAAC

(BG526387.1)
