# Supplementary material for: smRNAome profiling to identify conserved and novel microRNAs in Stevia rebaudiana Bertoni
Source: BMC Plant Biol. 2012 Nov 1;12:197. doi: 10.1186/1471-2229-12-197 (PMC3502355; doi:10.1186/1471-2229-12-197)
Supplement: Additional file 2 — Table S1. Target genes of conserved miRNAs identified using psRNATarget and TAPIR programs. [file 1471-2229-12-197-S2.doc]

**Supplementary Table 1: Target genes of conserved miRNAs identified using psRNATarget program and TAPIR.**

| **miRNA Acc.** | **Target Acc.** | **Program validation** | **score** | **mfe** | **start** | **seed**  **gap** | **Seed mismatch** | **Seed**  **gu** | **gap** | **Mismatch** | **gu** | **E** | **UPE** | **Annotation** | **E-value** | **Target**  **Inhibition** |
| --- | --- | --- | --- | --- | --- | --- | --- | --- | --- | --- | --- | --- | --- | --- | --- | --- |
| [miR156/157](http://plantgrn.noble.org/psRNATarget/getseq.do?sessionid=1341228357821127&source=srna&seqID=S1) | BG525285 | psRNA target and TAPIR | 4 | 0.71 | 447 | 0 | 0 | 0 | 0 | 4 | 0 | 3 | 11.768 | SBP-box protein [Torenia fournieri] | 1e- 42 | Cleavage |
|  | BG524211 | psRNA target and TAPIR | 4 | 0.71 | 362 | 0 | 0 | 0 | 0 | 4 | 0 | 3 | 12.231 | Squamosa promoter-binding protein, putative [Ricinus communis] | 2e-29 | Cleavage |
|  | BG522964 | TAPIR | 4 | 0.73 | 532 | 0 | 0 | 0 | 1 | 2 | 2 |  |  | zinc finger family protein [Arabidopsis lyrata] | 8e-06 |  |
| [miR160](http://plantgrn.noble.org/psRNATarget/getseq.do?sessionid=1341228357821127&source=srna&seqID=S15) | BG525867 | psRNA target and TAPIR | 4 | 0.74 | 283 | 0 | 0 | 0 | 0 | 4 | 0 | 2 | 21.484 | auxin response factor 10 [Solanum lycopersicum] | 6e-42 | Cleavage |
| miR166 | BG525793 | TAPIR | 3 | 0.83 | 39 | 0 | 1 | 0 | 0 | 1 | 0 |  |  | Glucan endo-1,3-beta-glucosidase precursor, putative [Ricinus communis] | 1e-11 | - |
| [miR167](http://plantgrn.noble.org/psRNATarget/getseq.do?sessionid=1341228357821127&source=srna&seqID=S43) | BG522436 | psRNA target | - | - | - | - | - | - | - | - | - | 3 | 10.772 | chloroplastic glutamine synthetase [Helianthus annuus] | 3e-72 | Cleavage |
|  | BG522386 | psRNA target | - | - | - | - | - | - | - | - | - | 3 | 16.234 | Early nodulin-like protein [Medicago truncatula] | 4e-70 | Cleavage |
| miR319 | BG525592 | TAPIR | 4 | 0.72 | 320 | 0 | 1 | 1 | 0 | 1 | 0 | - | - | catalase 3 [Helianthus annuus] | 8e-44 | - |
| miR393 | BG522349 | psRNA target | - | - | - | - | - | - | - | - | - | 2.5 | 12.932 | light harvesting chlorophyll a/b binding protein5 precursor [Zea mays] | 9e-112 | Cleavage |
|  | BG522409 | psRNA target | - | - | - | - | - | - | - | - | - | 2.5 | 13.451 | PREDICTED: chlorophyll a-b binding protein CP26, chloroplastic [Vitis vinifera] | 4e-109 | Cleavage |
|  | BG523298 | psRNA target | - | - | - | - | - | - | - | - | - | 2.5 | 13.492 | light harvesting chlorophyll a/b binding protein5 precursor [Zea mays] | 1e-82 | Cleavage |
|  | BG523498 | psRNA target | - | - | - | - | - | - | - | - | - | 2.5 | 13.613 | ASCAB9 [Dubautia arborea] | 7e-81 | Cleavage |
|  | BG524128 | psRNA target | - | - | - | - | - | - | - | - | - | 2.5 | 13.613 | ASCAB9 [Dubautia arborea] | 2e-74 | Cleavage |
|  | BG521481 | psRNA target | - | - | - | - | - | - | - | - |  | 2.5 | 13.613 | ASCAB9 [Dubautia arborea] | 2e-76 | Cleavage |
|  | BG523194 | psRNA target | - | - | - | - | - | - | - | - | - | 2.5 | 13.813 | ASCAB9 [Dubautia arborea] | 7e-81 | Cleavage |
| miR396 | BG526332 | TAPIR | 4 | 0.75 | 34 | 0 | 0 | 1 | 0 | 2 | 2 | - | - | peroxidase [Camellia oleifera] | 5e-113 | - |
|  | BG523664 | TAPIR | 4 | 0.75 | 213 | 0 | 0 | 1 | 0 | 2 | 2 | - | - | peroxidase [Camellia oleifera] | 2e-102 | - |
|  | BG524064 | TAPIR | 4 | 0.75 | 227 | 0 | 0 | 1 | 0 | 2 | 2 | - | - | secretory peroxidase [Nicotiana tabacum], | 5e-96 | - |
|  | BG526444 | TAPIR | 4 | 0.75 | 145 | 0 | 0 | 1 | 0 | 2 | 2 | - | - | peroxidase [Camellia oleifera] | 6e-111 | - |
|  | BG522375 | TAPIR | 4 | 0.75 | 271 | 0 | 0 | 1 | 0 | 2 | 2 | - | - | secretory peroxidase [Nicotiana tabacum], | 5e-97 | - |
|  | BG526288 | TAPIR | 4 | 0.75 | 322 | 0 | 0 | 1 | 0 | 2 | 2 | - | - | peroxidase [Cucumis melo] | 6e-108 | - |
|  | BG524152 | TAPIR | 4 | 0.75 | 263 | 0 | 0 | 1 | 0 | 2 | 2 | - | - | secretory peroxidase [Nicotiana tabacum] | 2e-103 | - |
|  | BG526227 | TAPIR | 4 | 0.75 | 287 | 0 | 0 | 1 | 0 | 2 | 2 | - | - | secretory peroxidase [Nicotiana tabacum] | 1e-92 | - |
|  | BG526657 | TAPIR | 4 | 0.75 | 322 | 0 | 0 | 1 | 0 | 2 | 2 | - | - | peroxidase [Cichorium intybus] | 2e-75 | - |
| miR408 | BG526054 | TAPIR | 3.5 | 0.77 | 176 | 0 | 0 | 1 | 0 | 2 | 1 | - | - | PREDICTED: uncharacterized protein LOC100832634 [Brachypodium distachyon] | 6e-06 | - |
| miR414 | BG523553 | TAPIR | 0 | 1 | 400 | 0 | 0 | 0 | 0 | 0 | 0 | - | - | no similarity |  | - |
|  | BG525537 | TAPIR | 0 | 1 | 261 | 0 | 0 | 0 | 0 | 0 | 0 | - | - | RING/U-box domain-containing protein [Arabidopsis thaliana] | 1e-34 | - |
|  | BG521371 | TAPIR | 0 | 1 | 432 | 0 | 0 | 0 | 0 | 0 | 0 | - | - | WD-repeat protein, putative [Ricinus communis] | 1e-81 | - |
|  | BG521415 | TAPIR | 0 | 1 | 597 | 0 | 0 | 0 | 0 | 0 | 0 | - | - | phytochrome E [Pieris nana] | 1e-73 | - |
|  | BG523266 | TAPIR | 0 | 1 | 706 | 0 | 0 | 0 | 0 | 0 | 0 | - | - | conserved hypothetical protein [Ricinus communis] | 5e-20 | - |
|  | BG526293 | TAPIR | 1 | 1 | 297 | 0 | 0 | 0 | 0 | 1 | 0 | - | - | unnamed protein product [Vitis vinifera] | 3e-09 | - |
|  | BG524976 | TAPIR | 1 | 0.87 | 575 | 0 | 0 | 0 | 0 | 1 | 0 | - | - | vacuolar ATP synthase subunit E, putative [Ricinus communis] | 1e-78 | - |
|  | BG526120 | TAPIR | 1 | 0.87 | 152 | 0 | 0 | 0 | 0 | 1 | 0 | - | - | PREDICTED: cyclin-P3-1-like isoform 1 [Vitis vinifera] | 5e-47 | - |
|  | BG525743 | TAPIR | 1 | 0.89 | 397 | 0 | 0 | 0 | 0 | 1 | 0 | - | - | unnamed protein product [Vitis vinifera] | 5e-12 | - |
|  | BG523220 | TAPIR | 2 | 0.93 | 8 | 0 | 1 | 0 | 0 | 0 | 0 | - | - | SIN3 component, histone deacetylase complex [Populus trichocarpa] | 6e-42 | - |
|  | BG521590 | TAPIR | 1 | 0.85 | 249 | 0 | 0 | 0 | 0 | 1 | 0 | - | - | putative ATP synthase [Arabidopsis thaliana] | 1e-14 | - |
|  | BG526602 | TAPIR | 1 | 0.89 | 285 | 0 | 0 | 0 | 0 | 1 | 0 | - | - | elongation factor 1-delta 1 [Arabidopsis thaliana] | 2e-31 | - |
|  | BG524292 | TAPIR | 1 | 0.85 | 423 | 0 | 0 | 0 | 0 | 1 | 0 | - | - | phosphatidylcholine transfer protein, putative [Ricinus communis] | 1e-28 | - |
|  | BG523324 | TAPIR | 1 | 0.85 | 453 | 0 | 0 | 0 | 0 | 1 | 0 | - | - | conserved hypothetical protein [Ricinus communis] | 2e-43 | - |
|  | BG521538 | TAPIR | 1 | 0.89 | 96 | 0 | 0 | 0 | 0 | 1 | 0 | - | - | elongation factor 1-beta [Zea mays] | 5e-15 | - |
|  | BG522106 | TAPIR | 2 | 0.89 | 499 | 0 | 1 | 0 | 0 | 0 | 0 | - | - | programmed cell death 2 C-terminal domain-containing protein [Arabidopsis thaliana] | 2e-32 | - |
|  | BG525199 | TAPIR | 2 | 0.87 | 474 | 0 | 0 | 0 | 0 | 2 | 0 | - | - | glycine-rich cell wall structural protein precursor [Zea mays] | 1e-05 | - |
|  | BG526710 | TAPIR | 2 | 0.87 | 630 | 0 | 0 | 0 | 0 | 2 | 0 | - | - | ALF domain class transcription factor [Malus x domestica] | 2e-79 | - |
|  | BG525022 | TAPIR | 2 | 0.89 | 541 | 0 | 0 | 0 | 0 | 2 | 0 | - | - | resistance protein RGC2 [Lactuca sativa] | 2e-35 | - |
|  | BG525945 | TAPIR | 2 | 0.89 | 547 | 0 | 0 | 0 | 0 | 2 | 0 | - | - | CWC15-like protein [Medicago truncatula] | 1e-59 | - |
|  | BG525676 | TAPIR | 3 | 0.76 | 273 | 0 | 1 | 0 | 0 | 1 | 0 | - | - | hypothetical protein VITISV_029430 [Vitis vinifera] | 5e-08 | - |
|  | BG526084 | TAPIR | 3 | 0.78 | 143 | 0 | 1 | 0 | 0 | 1 | 0 | - | - | nascent polypeptide associated complex alpha subunit, putative [Ricinus communis] | 3e-49 | - |
|  | BG522103 | TAPIR | 3 | 0.8 | 261 | 0 | 0 | 0 | 0 | 3 | 0 | - | - | serine/threonine protein phosphatase 2a regulatory subunit A,putative [Ricinus communis] | 5e-116 | - |
|  | BG525374 | TAPIR | 3 | 0.88 | 420 | 0 | 1 | 1 | 0 | 0 | 0 | - | - | PREDICTED: DUF21 domain-containing protein At1g47330 [Glycine max] | 1e-55 | - |
|  | BG522063 | TAPIR | 3 | 0.81 | 582 | 0 | 1 | 0 | 0 | 1 | 0 | - | - | zinc ion binding protein, putative [Ricinus communis] | 5e-17 | - |
|  | BG522373 | TAPIR | 1.5 | 0.97 | 117 | 0 | 0 | 1 | 0 | 0 | 1 | - | - | DNA-damage repair protein drt111, putative [Ricinus communis] | 2e-54 | - |
|  | BG522899 | TAPIR | 1.5 | 0.86 | 548 | 0 | 0 | 0 | 0 | 1 | 1 | - | - | PREDICTED: probable E3 ubiquitin-protein ligase makorin-1-like [Glycine max] | 7e-64 | - |
|  | BG522380 | TAPIR | 3 | 0.79 | 211 | 0 | 1 | 0 | 0 | 1 | 0 | - | - | Ribosome biogenesis protein BMS1-like protein [Medicago truncatula] | 4e-14 | - |
|  | BG522915 | TAPIR | 3 | 0.79 | 409 | 0 | 1 | 0 | 0 | 1 | 0 | - | - | PREDICTED: uncharacterized protein LOC100243695 [Vitis vinifera] | 4e-07 | - |
|  | BG523418 | TAPIR | 4 | 0.79 | 420 | 0 | 2 | 0 | 0 | 0 | 0 | - | - | HMG1/2-like protein [Glycine max] | 4e-25 | - |
|  | BG523106 | TAPIR | 3 | 0.79 | 674 | 0 | 1 | 0 | 0 | 1 | 0 | - | - | template-activating factor I [Arabidopsis thaliana] | 5e-90 | - |
|  | BG522175 | TAPIR | 4 | 0.77 | 301 | 0 | 1 | 0 | 0 | 2 | 0 | - | - | Ribosomal RNA adenine dimethylase family protein [Arabidopsis thaliana] | 1e-27 | - |
|  | BG522860 | TAPIR | 4 | 0.76 | 370 | 0 | 1 | 0 | 0 | 2 | 0 | - | - | potassium efflux antiporter [Populus trichocarpa] | 6e-10 | - |
|  | BG524583 | TAPIR | 4 | 0.74 | 154 | 0 | 1 | 0 | 0 | 2 | 0 | - | - | NO HIT |  | - |
|  | BG523653 | TAPIR | 4 | 0.77 | 127 | 0 | 1 | 0 | 0 | 2 | 0 | - | - | 60s acidic ribosomal protein-like protein [Solanum tuberosum] | 7e-25 | - |
|  | BG522394 | TAPIR | 4 | 0.73 | 304 | 0 | 1 | 0 | 0 | 2 | 0 | - | - | DNA polymerase lambda [Arabidopsis lyrata subsp. lyrata] | 6e-34 | - |
|  | BG524027 | TAPIR | 4 | 0.74 | 232 | 0 | 1 | 0 | 0 | 2 | 0 | - | - | hypothetical protein VITISV_007408 [Vitis vinifera] | 6e-06 | - |
|  | BG525422 | TAPIR | 3.5 | 0.87 | 379 | 0 | 1 | 0 | 0 | 1 | 1 | - | - | G-box binding factor [Medicago truncatula] | 2e-06 | - |
|  | BG522142 | TAPIR | 4 | 0.79 | 111 | 0 | 1 | 0 | 0 | 2 | 0 | - | - | alpha chain of nascent polypeptide associated complex [Nicotiana benthamiana | 1e-66 | - |
|  | BG525663 | TAPIR | 4 | 0.78 | 365 | 0 | 1 | 0 | 0 | 2 | 0 | - | - | predicted protein [Populus trichocarpa] | 9e-36 | - |
|  | BG524658 | TAPIR | 3 | 0.78 | 629 | 0 | 0 | 0 | 0 | 3 | 0 | - | - | tubulin alpha-2 chain [Populus trichocarpa] | 1e-93 | - |
|  | BG522826 | TAPIR | 4 | 0.88 | 309 | 0 | 1 | 1 | 0 | 1 | 0 | - | - | DEAD-box ATP-dependent RNA helicase 5 [Arabidopsis thaliana] | 7e-22 | - |
|  | BG523196 | TAPIR | 4 | 0.7 | 355 | 0 | 1 | 0 | 0 | 2 | 0 | - | - | co-chaperone protein SBA1 [Zea mays] | 7e-38 | - |
|  | BG525882 | TAPIR | 3 | 0.75 | 610 | 0 | 0 | 0 | 0 | 3 | 0 | - | - | SPX domain-containing protein 2 [Phaseolus vulgaris] | 2e-57 | - |
|  |  | TAPIR | 3 | 0.82 | 137 | 0 | 0 | 2 | 0 | 1 | 0 | - | - | membrane alanyl aminopeptidase [Arabidopsis lyrata subsp. lyrata],  TFIID component TAF2 [Arabidopsis thaliana] | 1e-10 | - |
|  | BG522158 | TAPIR | 3 | 0.86 | 277 | 0 | 0 | 2 | 0 | 1 | 0 | - | - | calmodulin [Elaeis oleifera] | 3e-58 | - |
|  | BG524561 | TAPIR | 4 | 0.77 | 366 | 0 | 1 | 1 | 0 | 1 | 0 | - | - | uncharacterized protein LOC100796372 [Glycine max] | 1e-44 | - |
|  | BG523925 | TAPIR | 3.5 | 0.79 | 105 | 0 | 1 | 0 | 0 | 1 | 1 | - | - | TIR-NBS-LRR class disease resistance protein [Arabidopsis thaliana] | 4e-05 | - |
|  | BG522631 | TAPIR | 4 | 0.76 | 287 | 0 | 1 | 1 | 0 | 1 | 0 | - | - | phosphoglucomutase [Triticum aestivum] | 2e-79 | - |
|  | BG521646 | TAPIR | 3 | 0.74 | 99 | 0 | 0 | 0 | 0 | 3 | 0 | - | - | NO HIT |  | - |
|  | BG523746 | TAPIR | 3.5 | 0.87 | 300 | 0 | 1 | 1 | 0 | 0 | 1 | - | - | o-linked n-acetylglucosamine transferase, ogt, putative [Ricinus communis] | 3e-30 | - |
|  | BG525206 | TAPIR | 3.5 | 0.72 | 460 | 0 | 1 | 0 | 0 | 1 | 1 | - | - | copper resistance D domain protein [Stenotrophomonas maltophilia] | 6e-08 | - |
|  | BG521837 | TAPIR | 4 | 0.76 | 91 | 0 | 1 | 1 | 0 | 1 | 0 | - | - | Phosphoglucomutase, cytoplasmic 2, putative, expressed [Oryza sativa Japonica Group] | 1e-74 | - |
|  | BG524432 | TAPIR | 3 | 0.77 | 453 | 0 | 0 | 1 | 0 | 2 | 0 | - | - | metal ion binding protein, putative [Ricinus communis] | 5e-22 | - |
|  | BG525207 | TAPIR | 3.5 | 0.72 | 460 | 0 | 1 | 0 | 0 | 1 | 1 | - | - | copper resistance D domain protein [Stenotrophomonas maltophilia] | 6e-08 | - |
|  | BG523805 | TAPIR | 3 | 0.86 | 383 | 0 | 0 | 2 | 0 | 1 | 0 | - | - | calmodulin 1 [Brassica oleracea] | 4e-85 | - |
|  | BG523705 | TAPIR | 4 | 0.76 | 66 | 0 | 0 | 1 | 0 | 1 | 0 | - | - | putative phosphoglucomutase [Amorphophallus konjac] | 3e-107 | - |
|  | BG525331 | TAPIR | 3 | 0.87 | 540 | 0 | 0 | 2 | 0 | 1 | 0 | - | - | molecular chaperone Hsp90-1 [Solanum lycopersicum] | 8e-68 | - |
|  | BG524208 | TAPIR | 4 | 0.77 | 612 | 0 | 1 | 1 | 0 | 1 | 0 | - | - | UDP-glycosyltransferase 73E1 [Stevia rebaudiana] | 2e-114 | - |
|  | BG523079 | TAPIR | 4 | 0.76 | 424 | 0 | 1 | 1 | 0 | 1 | 0 | - | - | Phosphoglucomutase, cytoplasmic 2, putative, expressed [Oryza sativa] | 1e-111 | - |
|  | BG524973 | TAPIR | 4 | 0.73 | 525 | 0 | 1 | 1 | 0 | 1 | 0 | - | - | PREDICTED: calmodulin-like protein 5-like [Vitis vinifera] | 9e-40 | - |
|  | AF097311 | TAPIR | 4 | 0.74 | 2297 | 0 | 1 | 0 | 0 | 2 | 0 | - | - | Stevia rebaudiana kaurene synthase (KS22-1) mRNA, complete cds | - | - |
|  | AF097310 | TAPIR | 4 | 0.74 | 2623 | 0 | 1 | 0 | 0 | 2 | 0 | - | - | Stevia rebaudiana calmodulin mRNA, complete cds | - | - |
|  | AF468661 | TAPIR | 3 | 0.86 | 387 | 0 | 0 | 2 | 0 | 1 | 0 | - | - | Stevia rebaudiana calmodulin mRNA, complete cds | - | - |
|  | AF474074 | TAPIR | 3 | 0.86 | 390 | 0 | 0 | 2 | 0 | 1 | 0 | - | - | Stevia rebaudiana calmodulin mRNA, complete cds | - | - |
|  | FB917637 | TAPIR | 4 | 0.77 | 1341 | 0 | 1 | 1 | 0 | 1 | 0 | - | - | Sequence 136910 from Patent WO2008034648 | - | - |
|  | HB787696. | TAPIR | 4 | 0.77 | 1341 | 0 | 1 | 1 | 0 | 1 | 0 | - | - | Sequence 136910 from Patent EP2090662 | - | - |
|  | AY345979 | TAPIR | 4 | 0.77 | 1341 | 0 | 1 | 1 | 0 | 1 | 0 | - | - | Stevia rebaudiana UDP-glycosyltransferase 73E1 mRNA, complete cds | - | - |
| miR856* | BG523449 | TAPIR | 3.5 | 0.8 | 16 | 0 | 1 | 0 | 0 | 0 | 3 | - | - | Elongation factor Tu [Arabidopsis thaliana] | 3e-82 | - |
| miR1310 | BG523172 | TAPIR | 4 | 0.73 | 213 | 0 | 1 | 0 | 1 | 1 | 0 | - | - | translation elongation factor 2 [Prunus persica] | 2e-114 | - |
